# *Codonopsis pilosula* polysaccharide attenuates the inflammatory response in macrophages induced by *Brucella* abortus outer membrane protein 19 via regulating *ATP2A1* to modulate cell adhesion and calcium signaling

**DOI:** 10.3389/fimmu.2026.1831002

**Published:** 2026-06-02

**Authors:** Xuxu Wang, Zhiyong Zhou, Nan Zhang, Ziying Zhang, Xingyue Qi, Xingguang Zhang, Zhiguo Gong, Wuzhi Zhong, Kun Liu, Yuan Shen

**Affiliations:** 1 Key Laboratory of Molecular Epidemiology of Chronic Diseases, School of Public Health, Inner Mongolia Medical University, Hohhot, China; 2 Xing’anmeng People’s Hospital, Wulanhot, Xing’an League, China; 3 School of Basic Medical Sciences, Inner Mongolia Medical University, Hohhot, China; 4 Laboratory of Veterinary Clinical Pharmacology, College of Veterinary Medicine, Inner Mongolia Agricultural University, Hohhot, China

**Keywords:** ATP2A1, Brucella outer membrane protein 19, calcium signaling, cell adhesion, Codonopsis pilosula polysaccharides, uterine damage

## Abstract

Brucellosis is a zoonotic disease caused by *Brucella* species. Its pathogenesis is closely associated with bacterial evasion of macrophage-mediated killing, induction of a pro-inflammatory cytokine storm, and immune-mediated pathological damage. Accumulating evidence indicates that *Codonopsis pilosula* polysaccharides (CPPS) inhibit inflammatory signaling, enhance macrophage phagocytosis, and exert immunomodulatory effects across multiple organs. However, the therapeutic potential of CPPS in brucellosis remains largely unexplored. This study aimed to investigate the effects of CPPS on the inflammatory response induced by *Brucella* outer membrane protein 19 (OMP19). *In vivo*, CPPS significantly alleviated tissue damage and simultaneously downregulated the expression of high mobility group box 1 protein (HMGB1), E-cadherin, and paxillin. *In vitro*, CPPS inhibited SYK/FAK/AKT phosphorylation, PKC activation, and WNT-1 signaling pathway transduction. Additionally, CPPS modulated the cytokine profile by downregulating pro-inflammatory cytokines (*TNF-α*, *IL-6*) while increasing the level of the anti-inflammatory cytokine IL-10. Furthermore, CPPS decreased the expression levels of E-cadherin and paxillin and reduced the intracellular calcium ion (Ca^2+^) concentration. *ATP2A1* was identified as a key differentially expressed gene through transcriptome sequencing. Knockdown experiments further confirmed that CPPS exerts anti-inflammatory effects by regulating *ATP2A1*. Collectively, CPPS attenuates the inflammatory response in macrophages induced by OMP19 via regulating *ATP2A1* to modulate cell adhesion and calcium signaling

## 1 Introduction

Brucellosis is a globally prevalent zoonotic chronic infectious disease caused by *Brucella* spp, clinically characterized by undulant fever, arthritis, reproductive system disorders (*e.g*., abortion, orchitis), and organ abscess formation (1). With more than 500,000 cases reported annually worldwide, the Inner Mongolia Autonomous Region of China is recognized as a high-risk endemic area, posing a significant public health threat that warrants urgent research attention (2–4). *Brucella* is a Gram-negative bacterium that is aerobic to microaerophilic and facultatively intracellular. Its pathogenicity mainly derives from its ability to evade host immune defenses and persist within phagocytes, such as macrophages and dendritic cells (DCs), as well as placental trophoblast cells (5, 6). Key virulence factors of *Brucella* include lipopolysaccharide (LPS), type IV secretion system (T4SS), and outer membrane proteins (OMPs). *Brucella* manipulates immune signaling by secreting effector proteins via the T4SS, which promotes adhesion, internalization, intracellular transport, and replication within host cells (7–9). Among these factors, OMPs play multifaceted roles in pathogenesis: (i) mediating adhesion to host cells and facilitating intracellular replication, and (ii) modulating host immune responses to promote immune evasion and persistent infection (10, 11). Among them, OMP19 is highly conserved, and lipidated OMP19 is consistently expressed in all *Brucella* species (12). Studies have shown that OMP19 can significantly alter the secretion patterns of cytokines and chemokines (13), inducing markedly higher *TNF-α* and *IL-6* production compared with OMP10 and OMP28, both *in vitro* and *in vivo* (14).

Adhesion of *Brucella* to host cells is a pivotal step in infection, with specific molecular interactions determining bacterial intracellular fate and influencing disease progression (15). This process triggers tyrosine phosphorylation of adhesome proteins such as focal adhesion kinase (FAK), Src kinase, paxillin, and p130Cas, which are integral to the integrin signaling pathway and regulate cell adhesion, migration, and proliferation (16). FAK enhances phagocytosis by coordinating macrophage migratory behavior and adhesion turnover, thereby facilitating target engulfment (17, 18). Protein kinase C (PKC), a family of serine/threonine kinases, participates in diverse signaling pathways related to cell proliferation, differentiation, and transcriptional regulation, and certain isoforms also regulate adhesion and migration (19). Classical PKC activation requires synergistic calcium ion (Ca^2+^) and diacylglycerol (DAG) signaling (20). Similarly, the WNT-1 signaling pathway stabilizes β-catenin binding to adhesion proteins, strengthens intercellular junctions, and influences cytoskeletal reorganization (21, 22). Existing studies have confirmed that *ATP2A1* is simultaneously involved in the regulation of cell adhesion and calcium signaling pathways (23).

Macrophages, as central effectors of innate immunity, are both the primary target cells of *Brucella* and critical regulators of host defense. They eliminate pathogens through adhesion molecule-mediated migration and phagocytosis and activate adaptive immunity via antigen presentation (24–26). Following invasion through the skin, mucosa, or gastrointestinal tract, *Brucella* are phagocytosed by local macrophages, which subsequently migrate to regional lymph nodes to establish primary foci of infection (5). *Brucella* evade immune clearance by interacting with macrophage surface receptors via lipid rafts, thereby inhibiting phagocytosis and preventing phagolysosomal fusion (8).

In recent years, immunomodulatory compounds derived from traditional Chinese medicine (TCM) have emerged as promising strategies for combating intracellular bacterial infections. CPPS, the primary bioactive components of *Codonopsis pilosula*, exhibit multiple immunomodulatory properties (27). CPPS modulate cytokine secretion, inhibit MAPK and NF-κB activation, enhance macrophage phagocytosis and bacterial killing, and suppress the growth of *Escherichia coli* (*E. coli*) (28). They also promote *IL-1β* secretion from macrophages and elevate serum *IL-1β* levels in mice, exerting immune-enhancing effects (29). Moreover, CPPS ameliorates inflammation in both the intestine and lungs, as demonstrated by improved phagocytic function of mouse alveolar macrophages in COPD models, reduced *IL-6*, *IL-8*, and *TNF-α* levels, and improved systemic inflammatory status (30). CPPS also protects immune organs from injury in cyclophosphamide-induced immunosuppression models (31). Collectively, these findings indicate that CPPS restores macrophage function and protects immune organs, demonstrating broad immunomodulatory capabilities. However, whether CPPS exerts similar effects in *Brucella* infection-induced inflammatory responses remains unknown. Therefore, this study aimed to investigate the role of CPPS in OMP19-induced inflammatory responses using cell and mouse models of *Brucella* infection.

## 2 Materials and methods

### 2.1 Experimental animals

Thirty-six female BALB/C mice (6–8 weeks old, 20–22 g) were purchased from SPF Biotechnology Spectrum Laboratory Animal Center (Beijing, China). Animals were housed at 22 ± 2 °C under specific pathogen-free conditions with a 12 h light/dark cycle and were provided autoclaved food and purified water *ad libitum*. All experimental procedures were approved by the Animal Ethics Committee of Inner Mongolia Medical University (approval ID: YKD202503042).

### 2.2 Mice model

Thirty-six female BALB/C mice (6–8 weeks old, 20–22 g) were randomly assigned to six groups (n = 6 mice per group) [unst: control, CPPS-treated groups (100 and 300 mg·kg^-1^), OMP19-treated group (5 mg·kg^-1^), and CPPS+OMP19-treated groups (100 and 300 mg·kg^-1^ CPPS were pre-injected intraperitoneally for 1 h, followed by 5 mg·kg^-1^ OMP19)], whereas mice in the control group were treated with PBS. After one week, pentobarbital sodium was administered via intraperitoneal injection at a dose of 150–200 mg·kg^-1^ (prepared as a 1% w/v solution; 0.3-0.4 ml was injected for a 20 g mouse). Following deep anesthesia, euthanasia was performed by cervical dislocation. Uterine tissues were collected and preserved in 4% paraformaldehyde, with a portion stored at -80 °C. The concentration of CPPS used in this study was based on previous studies (28, 32, 33).

### 2.3 OMP19 extraction and purification method

The *Brucella* OMP19 gene (GenBank: U35742) was synthesized (Gene-Optimal, Shanghai, China) and cloned into the pET-28a vector (34). The recombinant plasmid pET-28a-OMP19 was transformed into *E. coli* BL21 (DE3) by heat shock (42 °C, 90 s). Transformants were inoculated at a 1:100 dilution into LB medium containing 100 μg/ml kanamycin (MedChemExpress, USA) and cultured at 37 °C with shaking until OD_600_ reached 0.6 (35). Recombinant OMP19 expression was induced with 1 mM isopropyl β-D-1-thiogalactopyranoside (IPTG, MedChemExpress, USA) at 20 °C for 20 h with shaking.

Cells were harvested and lysed in buffer (60 mM sodium phosphate, 500 mM NaCl, 20 mM imidazole, pH 8.0), followed by sonication in an ice bath (250 W, 10 s on/10 s off, total 4 min). The lysate was centrifuged at 8,000 × g for 45 min at 4 °C, and the His-tagged OMP19 in the supernatant was purified by immobilized metal affinity chromatography (IMAC) using a 1 ml Ni-NTA Superflow cartridge (Bestchrom, Shanghai, China), which was equilibrated and washed with the same lysis buffer.

Protein concentration and characterization were confirmed by A_280_ quantification, 12% SDS-PAGE, and Western blotting with HRP-conjugated anti-His Tag monoclonal antibodies.

### 2.4 Transcriptome sequencing and analysis

#### 2.4.1 RNA extraction, library construction, and sequencing

Peritoneal macrophages isolated from BALB/C mice were pretreated with CPPS (Solarbio, SR8560, China) for 12 h and subsequently stimulated with OMP19 for 12 h. Total RNA was extracted using TRIzol reagent and assessed using Qubit 3.0 and Agilent 5300 (RIN > 7.0). Two micrograms of RNA were purified, fragmented, and reverse transcribed into double-stranded cDNA. Following end repair, A-tailing, adapter ligation, and PCR amplification, a strand-specific library with an insert size of 400 ± 50 bp was constructed and sequenced on the NovaSeq™ X Plus platform with 150 bp paired-end reads.

#### 2.4.2 Bioinformatics analysis

Raw reads were filtered to remove low-quality sequences and adapter contamination using Cutadapt (v1.9). High-quality, clean data were obtained after quality control using FastQC (v0.11.9) and deposited in the NCBI GEO/SRA database.

### 2.5 Cell extraction and cultivation

Three days before the isolation of peritoneal macrophages, BALB/C mice were intraperitoneally injected with 2 mL of 3% thioglycolate medium (BD Biosciences, 225620, Sparks, MD, USA). Peritoneal macrophages were collected by lavage of the peritoneal cavity with endotoxin-free phosphate-buffered saline (PBS) (Servicebio, G4202, China), followed by centrifugation at 2376 × g for 5 min at 4 °C. The cells were cultured at 37 °C in 5% CO_2_ in RPMI 1640 medium (Gibco, C11875500BT, China) supplemented with 10% fetal bovine serum (FBS) (ExCell Bio, FSD500, China). The cells (2 × 10^6^ cells per well) were seeded into 6-well culture plates in 1 mL of fresh culture medium and washed three times with PBS before infection or stimulation. The isolation and purification procedures of macrophages from peritoneal lavage fluid were performed strictly according to the experimental methods described in the cited literature. No additional cell sorting or special purification techniques were adopted in this study (36).

### 2.6 Western blotting

Mouse peritoneal macrophages were treated with 50 µg·mL^-1^ CPPS for 12 h, followed by stimulation with 5 µg·mL^-1^ OMP19 for 8 h. Total protein was extracted using cell lysis buffer (Servicebio, G2002, China) supplemented with phosphatase inhibitors (Beyotime, P1045, China). The cells were resuspended in 50 µL of lysis buffer, incubated on ice for 5 min, scraped, and centrifuged at 13,684 × g for 10 min at 4 °C.

A total of 40 µg of protein was quantified using the Enhanced BCA Protein Assay kit (Beyotime, P0398L, China) and separated by 12% SDS-PAGE (Solarbio, P1204, China). The proteins were transferred to PVDF membranes (Immobilon, ISEQ00010, Germany) using a Bio-Rad electroblotting system. The membranes were blocked with 5% non-fat milk (Becton, Dickinson and Company, 232100, USA) for 3.5 h at room temperature. Primary antibodies against *β-actin* (Affinity, Cat. No. AF7018, China), AKT (Proteintech, 10176-2-AP, China), SYK (Proteintech, 14858-1-AP, China), FAK (Affinity, AF6397, China), ERK (Affinity, AF0155, China), PKC (Affinity, AF6197, China), WNT-1 (Proteintech, 27935-1-AP, China), paxillin (Affinity, AF6332, China), E-cadherin (Affinity, BF0219, China), phospho-AKT (P-AKT) (Cell Signaling Technology, 4060T, USA), phospho-ERK (P-ERK) (Affinity, AF1015, USA), phospho-SYK (P-SYK) (Cell Signaling Technology, 2710T, USA), and phospho-FAK (P-FAK) (Affinity, AF3398, USA) were used.

Primary antibodies were diluted as follows: AKT at 1:3000, *β-actin* at 1:3000, and all other antibodies at 1:1000, using the antibody dilution buffer provided by Beyotime (China). The membranes were incubated with primary antibodies overnight at 4 °C and washed three times with TBST for 10 min each. After washing, the membranes were incubated with a secondary antibody (Affinity, S0001, China) at a dilution of 1:3000 for 1 h, followed by three additional washes with TBST, each for 10 min. Protein bands were visualized using enhanced chemiluminescence (Beyotime, P0018S, China) on PVDF membranes. All Western blot experiments were explicitly repeated independently three times.

### 2.7 Quantitative real-time PCR

Cell RNA extractions followed the Axygen RNA kit manufacturer’s instructions (Axygen Scientific, UE-MN-MS-RNA-250, USA). Cells were resuspended in lysis buffer and centrifuged at 13,684 × g, followed by RNA extraction as per the manufacturer’s instructions (A260/A280 value in the range of 1.9-2.0). The extracted RNA was reverse transcribed into cDNA using the PrimeScript™ RT Master Mix Kit (Vazyme, R223-01, China). The expression levels of *TNF-α*, *IL-6*, *IL-10*, *CXCL-10*, *MIP-1α*, and *CD40* relative to *β-actin* mRNA were measured using qPCR. Primers were designed based on sequence information obtained from NCBI (National Center for Biotechnology Information) and are listed in **Table 1**. The primers were synthesized by Sangon. For qPCR, the following conditions were used: initial denaturation at 95 °C for 10 min, followed by 40 cycles of denaturation at 95 °C for 15 s and annealing/extension at 60 °C for 30 s using the ABI QuantStudio™ 7 system. The qPCR procedures adhered to MIQE (Minimum Information for Publication of Quantitative Real-Time PCR Experiments) standards. To reduce variation between datasets, the arithmetic mean was calculated for the control and treatment groups, with three parallel samples per group, and the relative quantification (RQ) values were determined using the 2^-ΔΔCt^ method. All qPCR experiments were explicitly repeated independently three times.

**Table 1:** Primer messages of qPCR.

Primer name	Forward (5’-3′)	Reverse (5′-3′)	Gen Bank accession No.
*TNF-α*	CTTCTCATTCCTGCTTGTG	ACTTGGTGGTTTGCTACG	XM_021163844.1
*IL-6*	TTCTTGGGACTGATGCTG	CTGGCTTTGTCTTTCTTGTT	XM_021149735.1
*IL-10*	GGTTGCCAAGCCTTATCGTA	ACCTGCTCCACTGCCTTGCT	NM_010548.2
*CXCL10*	CAGTGAGAATGAGGGCCATAGG	CGGATTCAGACATCTCTGCTCAT	XM_021161764.2
*CD40*	ACACAGGTAGAAACCCCAGG	GTAATGTACCCCGTGTGTGC	NM_170702.2
*MIP-1α*	GCTCCCAGCCAGGTGTCATTTT	AAGACTCTCAGGCATTCAGTTCCAG	NM_011337.2
*β-actin*	CGTTGACATCCGTAAAGACC	TAGGAGCCAGAGCAGTAATC	NM_007393.5

### 2.8 Hematoxylin-eosin staining

Female BALB/C mice were randomly divided into 6 groups (n = 6 per group): control group, CPPS alone treatment groups (100 and 300 mg·kg^-1^), OMP19 model group (5 mg·kg^-1^), and CPPS pretreatment combined with OMP19 groups (intraperitoneal injection of 100 or 300 mg·kg^-1^ CPPS 1 h in advance, followed by administration of 5 mg·kg^-1^ OMP19). Mice in the control group received an equal volume of PBS via intraperitoneal injection. After one week, mice were euthanized, and uterine tissues were harvested. Tissues were fixed overnight in 4% paraformaldehyde at 4 °C, dehydrated in a graded ethanol series, embedded in paraffin, and sectioned at 6 μm. Sections were stained with H&E. Pathological alterations in uterine tissues were examined using an Axio Scan Z1 slide scanner (Zeiss, Germany). Four high-magnification fields of view were selected for each H&E-stained section.

At present, there is no universally recognized and unified histological scoring or grading system specifically applied to endometrial tissues in pathological studies. Therefore, a self-established semi-quantitative grading standard was adopted in this study to comprehensively evaluate the histopathological changes of the endometrium. All evaluation indicators were formulated based on the typical pathological injury characteristics of uterine tissues and included three core observation dimensions. First, the structural integrity of endometrial luminal epithelial cells was observed under a high-power microscope, and the occurrence and severity of cellular vacuolar degeneration were evaluated. Second, the structural integrity of glandular epithelial cells in both the functional layer and basal layer of the endometrium was synchronously assessed, and the positive rate and distribution range of vacuolar degeneration in glandular epithelial cells were statistically analyzed. Third, the infiltration degree of inflammatory immune cells in the endometrial stroma was quantitatively determined, including macrophages, eosinophils, neutrophils, basophils, and lymphocytes. Notably, no obvious inflammatory immune cell infiltration was detected in the blank control group. Accordingly, the evaluation criterion for elevated immune cell infiltration was defined as the histologically visible presence of infiltrated immune cells relative to the blank control.

### 2.9 Immunofluorescence assay

Mouse uterine tissues were embedded in paraffin and cut into 6 μm sections. Antigen retrieval was performed by heating the sections in 0.01 M citrate buffer (pH 6.0) at 95-98 °C for 20 min, followed by natural cooling to room temperature. Thereafter, sections were deparaffinized in xylene, rehydrated through a graded ethanol series, and rinsed twice with pre-chilled PBS (4 °C) for 5 min each. Sections were blocked with 3% bovine serum albumin (BSA) in PBS at room temperature for 4 h. Subsequently, sections were incubated overnight at 4 °C in the dark with HMGB1 (Affinity, AF7020, China) primary antibody diluted 1:100 in PBS containing 3% BSA. After incubation, sections were washed twice with PBST for 10 min each, then incubated with Alexa Fluor 488-conjugated secondary antibody (Affinity, S0018, China) at room temperature for 1 h, followed by three washes with PBST for 5 min each. Finally, all samples were observed and imaged using a ZEISS confocal microscope under identical imaging parameters, and grayscale distribution values were analyzed accordingly.

### 2.10 Macrophage intracellular Ca^2+^ detection

Mouse primary peritoneal macrophages were cultured in serum-free medium using 6-well plates at a concentration of 2 × 10^6^ cells. Macrophages were pretreated with CPPS (50 µg·mL^-1^) for 12 h, followed by stimulation with OMP19 (5 µg·mL^-1^) for an additional 12 h. Subsequently, the culture medium was aspirated, and the cells were gently rinsed once with PBS. Fluo-4 staining solution (1 mL per well in 6-well plates) (Beyotime, S1061S, China) was applied to cover the cell monolayer. Incubation was performed for 30 min at 37 °C, protected from light. The cells were then washed 1–3 times with PBS or HBSS after incubation. Fluorescence signals were quantified using an inverted fluorescence microscope (Fluo-4 AM emits green fluorescence, Ex/Em = 490/525 nm) with consistent exposure settings across samples.

### 2.11 Immunohistochemistry in uterine tissue

The expression levels of E-cadherin and paxillin in mouse endometrial tissue were detected by IHC (n = 6 per group). Briefly, sections were deparaffinized, rehydrated, and subjected to antigen retrieval, followed by blocking with normal serum. The samples were then incubated overnight at 4 °C with primary antibodies against E-cadherin and paxillin (1:200). After washing, the sections were incubated with appropriate secondary antibodies (1:500) (Affinity, S0001, China) at room temperature for 1 h. Immunoreactivity was visualized using a DAB chromogenic substrate, and nuclei were counterstained with hematoxylin. The signal intensity of yellow staining in mouse endometrial tissue was quantified using the H-score calculation software integrated into the Aipathwell platform.

### 2.12 The calcium ion chelator BAPTA-AM

Following a 1 h pretreatment with 100 µM BAPTA-AM (MCE, HY-100545, China), mouse peritoneal macrophages were sequentially treated with 50 µg·mL^-1^ CPPS for 12 h and stimulated with 5 µg·mL^-1^ OMP19 for 8 h. The expression level of E-cadherin protein was then determined using Western blot analysis.

### 2.13 Culture of THP-1 cells and *ATP2A1* gene knockdown

THP-1 cells were seeded into 6-well plates at a density of 2 × 10^6^ cells/mL, and siRNA transfection was performed after cell adherence. siRNA (Sangon Biotech, 113195091, China) and Lipofectamine 2000 (Thermo Fisher Scientific, 11668019, USA) were diluted separately with Opti-MEM medium (Thermo Fisher Scientific, 31985070, USA), incubated at room temperature for 5 min, then mixed thoroughly and incubated for an additional 20 min to form transfection complexes. The culture medium was replaced with Opti-MEM, and the complexes were added to a final volume of 2 mL. After 24 h of incubation, the medium was replaced with complete medium for subsequent treatment at a final concentration of 20 nmol/L.

### 2.14 Data analysis

Data are expressed as the mean ± standard deviation (mean ± SD). Statistical analyses were performed using GraphPad Prism 9. The normality of distribution was assessed by the Shapiro-Wilk test, and homogeneity of variance was evaluated using the Brown-Forsythe test. One-way or two-way analysis of variance (ANOVA) was applied for comparisons among multiple groups, followed by Bonferroni or Tukey *post hoc* multiple comparison tests as indicated in the figure legends; non-normally distributed data were analyzed using the non-parametric Kruskal-Wallis test. Effect size (Cohen’s d) was calculated for key pairwise comparisons. A *P* value ≤ 0.05 was considered statistically significant.

## 3 Results

### 3.1 Effects of CPPS on OMP19-induced uterine inflammation and HMGB1 expression

**Figure 1:**
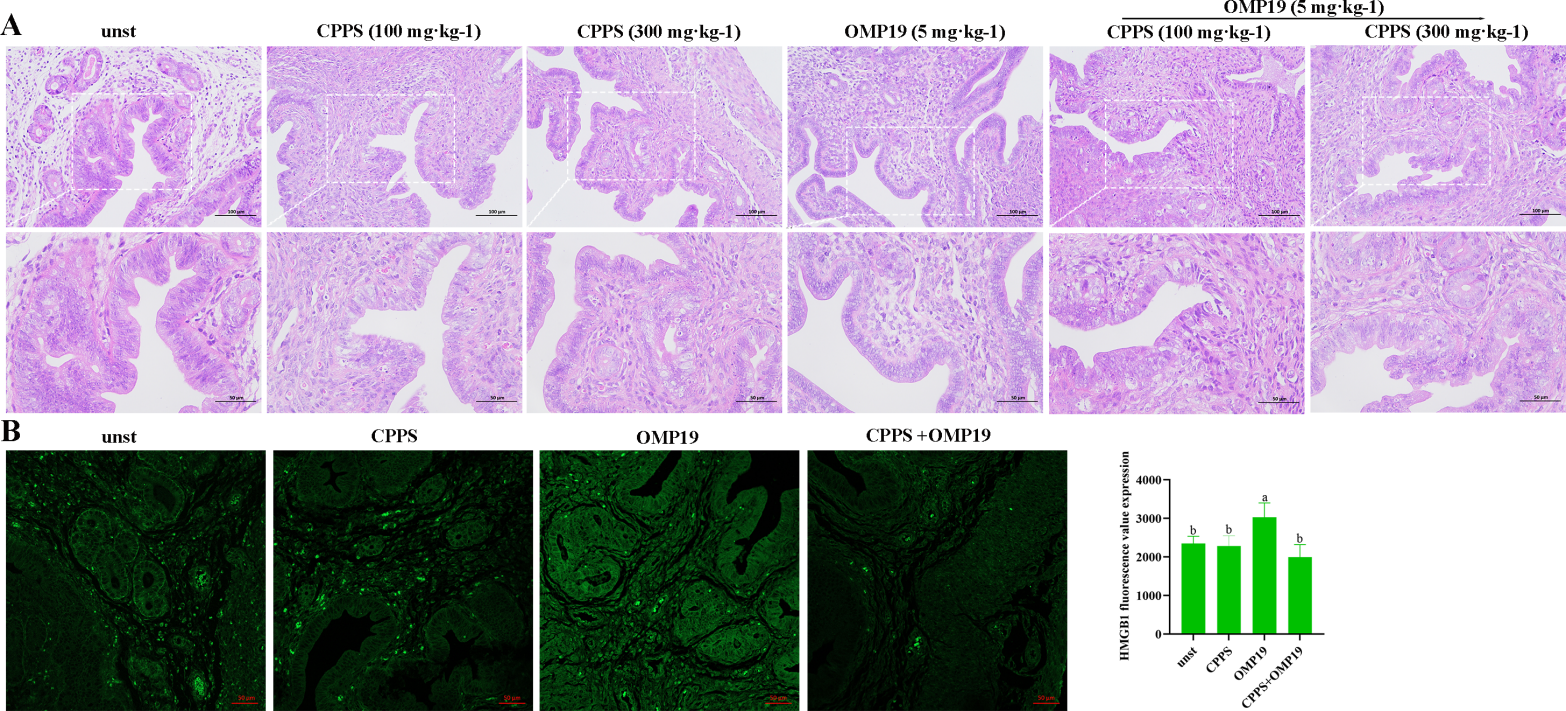
Effects of CPPS on OMP19-induced uterine inflammation and HMGB1 expression. **(A)** Mice were pretreated with different concentrations of CPPS (100 or 300 mg·kg^-1^ for 1 h via intraperitoneal injection) and then induced with OMP19 (5 mg·kg^-1^). Tissue sections were stained with H&E (one week post-induction). Scale bar = 50 μm or 100 μm. Three independent experiments were performed. **(B)** CPPS (100 mg·kg^-1^) reduced OMP19-induced HMGB1 expression in the mouse uterus. HMGB1 protein expression (green) was analyzed by microscopy (one week post-induction, ×100 magnification). Fluorescence intensity was quantified using Zen software (Zeiss). Cohen’s d = 2.97 for comparison between OMP19 and CPPS+OMP19 groups (extremely large effect). n = 6. Lowercase letters a, b, c and d in the figures indicate statistical significance. Different letters mean significant differences between groups, while the same letter indicates no significant difference.

The inhibitory effects of CPPS on inflammation were investigated *in vivo*. Mice were preconditioned with CPPS (at doses of 100 or 300 mg·kg^-1^ body weight; n = 6) via intraperitoneal injection; after 1 h, they were treated with an intraperitoneal dose of OMP19 (5 mg·kg^-1^). OMP19 exposure induced uterine epithelial cell vacuolar degeneration and prominent neutrophil infiltration. Both doses of CPPS (100 mg·kg^-1^ and 300 mg·kg^-1^) reduced the extent of this blister-like degeneration in uterine epithelial cells and decreased the number of neutrophils induced by OMP19 (**Figure 1A**). To assess CPPS-mediated tissue repair, uterine HMGB1 expression was analyzed. We showed that CPPS significantly suppressed OMP19-induced HMGB1 upregulation (*P* < 0.001, **Figure 1B**), suggesting its role in modulating HMGB1-associated inflammatory responses and tissue damage.

### 3.2 Transcriptome analysis

**Figure 2:**
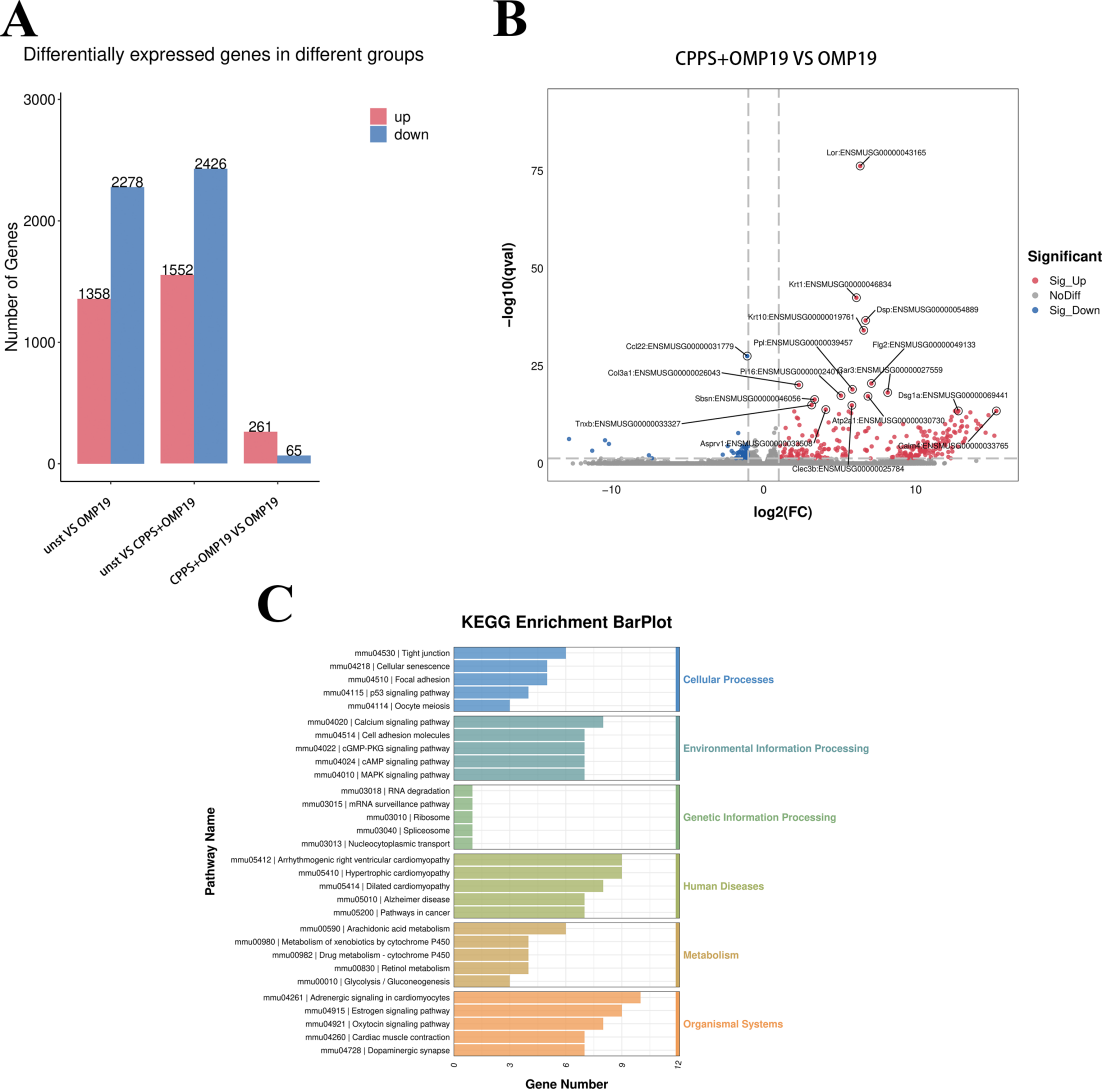
Results of RNA-seq analysis of mouse peritoneal macrophages. Mouse peritoneal macrophages were pretreated with 50 µg·mL^-1^ CPPS for 12 h, followed by 5 µg·mL^-1^ OMP19 for 12 h. **(A)** Differentially expressed genes between the CPPS+OMP19 co-treatment group and the OMP19 group. A total of 261 upregulated and 65 downregulated genes were identified. **(B)** Volcano plot showing the top 20 differentially expressed genes. **(C)** KEGG enrichment analysis showing that the differentially expressed genes were mainly enriched in cell adhesion and Ca^2+^ signaling pathways.

To elucidate the mechanism by which CPPS alleviates OMP19-induced tissue injury and inflammation, mouse peritoneal macrophages were used in this study, and differentially expressed genes were screened and analyzed via transcriptome sequencing (RNA-seq). Transcriptomic profiling revealed 261 upregulated and 65 downregulated genes in the CPPS+OMP19 group compared to the OMP19 group (**Figure 2A**). KEGG pathway analysis of differentially expressed genes (DEGs, **Figure 2C**) identified significant enrichment in cellular adhesion pathways (*e.g*., focal adhesion including SYK FAK, AKT signaling) and calcium signaling (*e.g*., Ca^2+^ influx regulators, including ERK, PKC, WNT-1). Among the top 20 DEGs (**Figure 2B**), we further screened and identified *ATP2A1* as the core upstream regulatory gene. This gene is involved in both cell adhesion and Ca²^+^ influx, and was therefore selected for further in-depth investigation.

### 3.3 Effect of CPPS on the expression of inflammatory cytokines, chemokines, and macrophage activation in OMP19-induced macrophages

**Figure 3:**
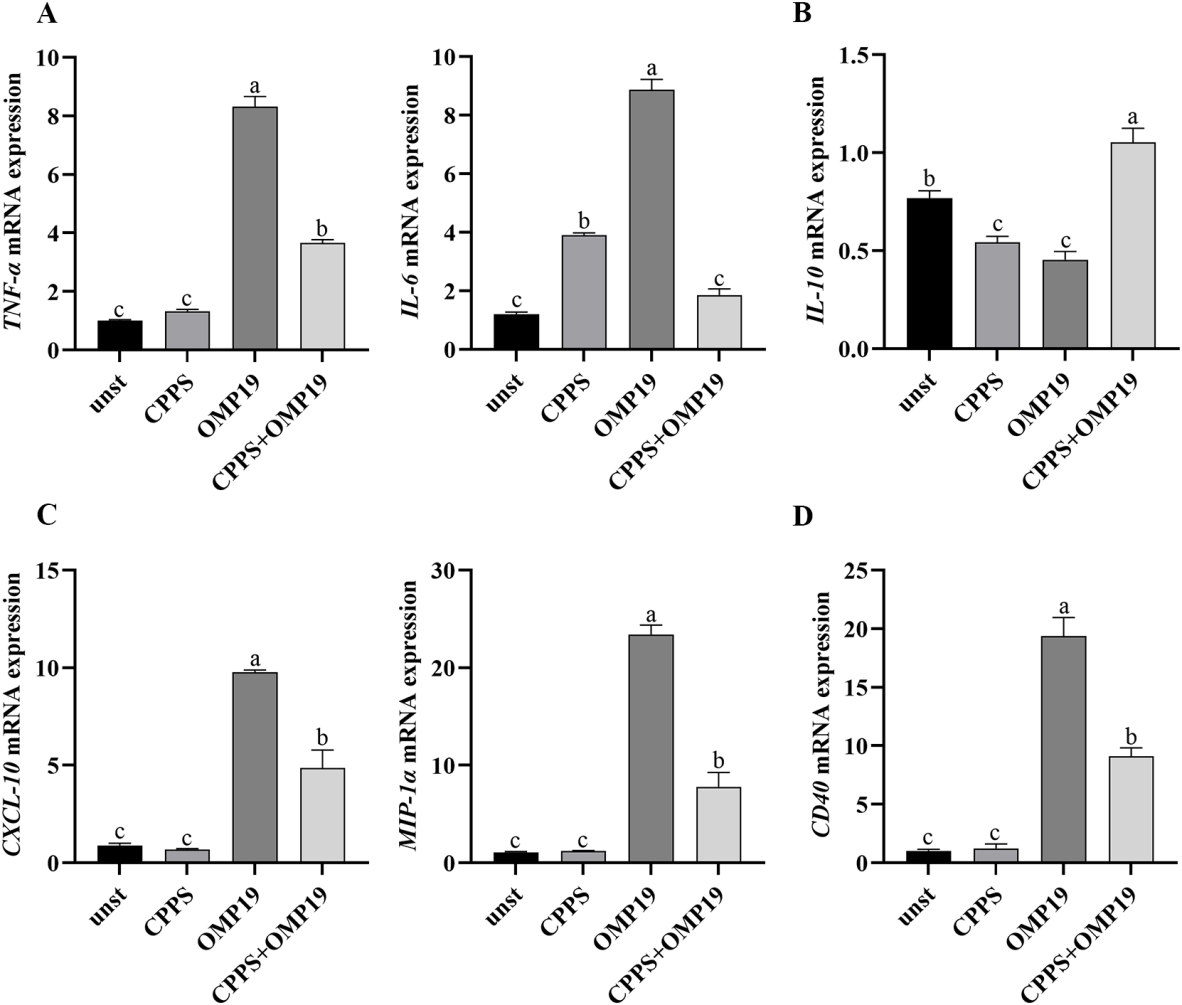
Effect of CPPS on the expression of inflammatory cytokines, chemokines, and macrophage activation in macrophages induced by OMP19. Mouse peritoneal macrophages were pretreated with 50 µg·mL^-1^ CPPS for 12 h, followed by 5 µg·mL^-1^ OMP19 for 12 h. mRNA expression levels were investigated using RT-qPCR. **(A)** *TNF-α*, *IL-6*, **(B)** *IL-10*, **(C)** *CXCL-10*, *MIP-1α*, **(D)** *CD40*. n = 3. Lowercase letters a, b, c and d in the figures indicate statistical significance. Different letters mean significant differences between groups, while the same letter indicates no significant difference.

To elucidate the immunomodulatory effects of CPPS in macrophages induced by OMP19, we examined the expression levels of inflammatory cytokines (*TNF-α*, *IL-6*, and *IL-10*), chemokines (*MIP-1α* and *CXCL-10*), and co-stimulatory molecules (*CD40*) using RT-qPCR. Our results showed that OMP19 significantly induced the expression of inflammation-related cytokines *TNF-α*, *IL-6*, *CXCL-10*, *MIP-1α*, and *CD40* in macrophages compared with the control group (*P* < 0.001). In contrast, CPPS significantly suppressed this OMP19-induced upregulation (*P* < 0.001, **Figures 3A, C, D**). In addition, OMP19 significantly decreased the expression of the anti-inflammatory cytokine *IL-10* in macrophages (*P* < 0.001), whereas CPPS upregulated the OMP19-induced expression of *IL-10* (*P* < 0.05, **Figure 3B**). These results suggest that CPPS may inhibit excessive macrophage activation, thereby alleviating inflammatory responses.

### 3.4 CPPS down-regulated the expression of E-cadherin and paxillin in OMP19-induced endometrial tissue

**Figure 4:**
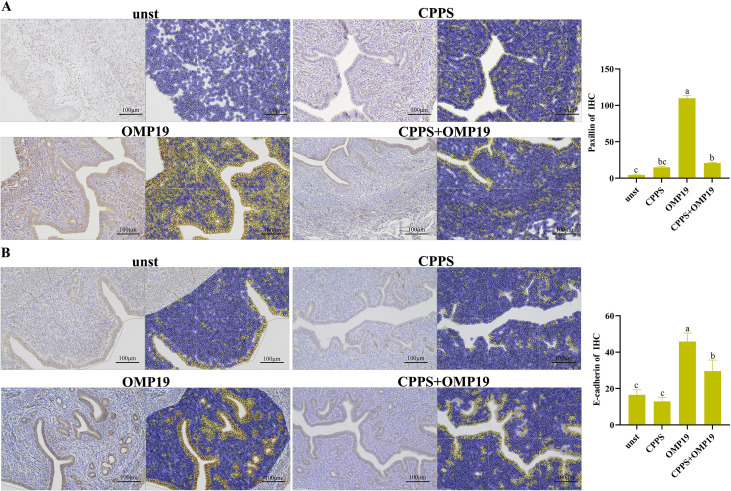
CPPS downregulated the expression of E-cadherin and paxillin in OMP19-induced endometrial tissue. Mice were pretreated with CPPS (100 mg·kg^-1^, intraperitoneal injection) for 1 h, followed by induction with OMP19 (5 mg·kg^-1^). Indicators were measured one week post-induction. Tissue sections were subjected to IHC to detect **(A)** paxillin and **(B)** E-cadherin expression levels in mouse endometrial tissue. The positive signal intensity in endometrial tissue was quantified using the H-score module integrated into the Aipathwell software, and the mean value was used for statistical analysis. Paxillin: Cohen’s d = 16.78 between OMP19 and CPPS+OMP19 groups (extremely large effect). For E-cadherin, Cohen’s d = 2.99 between OMP19 and CPPS+OMP19 groups (extremely large effect). n = 6.

To further elucidate the expression levels and localization of E-cadherin and paxillin, immunohistochemical (IHC) analysis was performed on mouse endometrial tissue. As shown in **Figure 4**, the results demonstrated specific expression of E-cadherin and paxillin in both luminal and glandular epithelial cells of the endometrium, with positive signals indicated by yellow staining. Notably, OMP19 treatment significantly upregulated the expression of both E-cadherin and paxillin (*P* < 0.05). In contrast, CPPS administration markedly reduced the expression of E-cadherin and paxillin in mouse endometrial tissue.

### 3.5 CPPS down-regulated the expression of E-cadherin and paxillin in macrophages induced by OMP19

**Figure 5:**
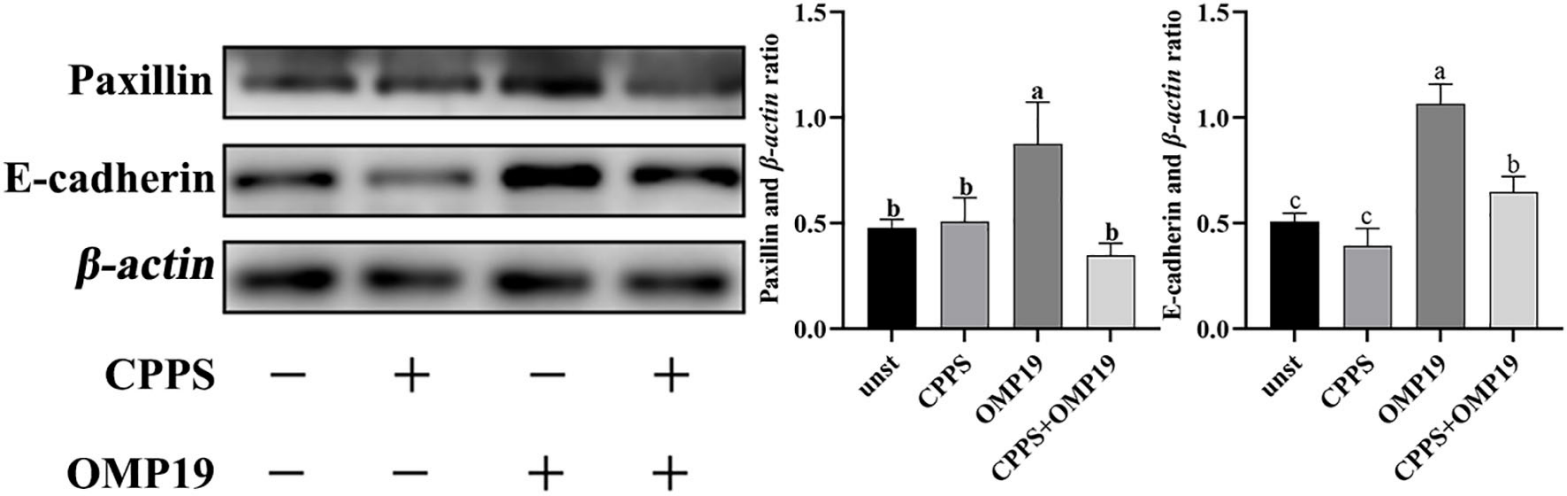
CPPS downregulated the expression of E-cadherin and paxillin in macrophages induced by OMP19. Mouse peritoneal macrophages were pretreated with 50 µg·mL^-1^ CPPS for 12 h, followed by 5 µg·mL^-1^ OMP19 for 8 h. E-cadherin and paxillin expression levels were measured using Western blot. *β-actin* served as a loading control. Grayscale values were measured using ImageJ software. n = 3.

To investigate the potential effects of CPPS on OMP19-mediated regulation of macrophage adhesion molecule expression, E-cadherin and paxillin protein levels were analyzed by Western blot. As shown in **Figure 5**, OMP19 stimulation significantly upregulated the expression levels of E-cadherin and paxillin in macrophages (*P* < 0.001), demonstrating its role in promoting macrophage adhesive capacity. In contrast, CPPS treatment markedly attenuated the OMP19-induced upregulation of these adhesion molecules compared with OMP19 alone (*P* < 0.001). These data indicate that CPPS counteracts OMP19-mediated enhancement of macrophage adhesion.

### 3.6 CPPS inhibited the inward flow of Ca^2+^ in macrophages induced by OMP19

**Figure 6:**
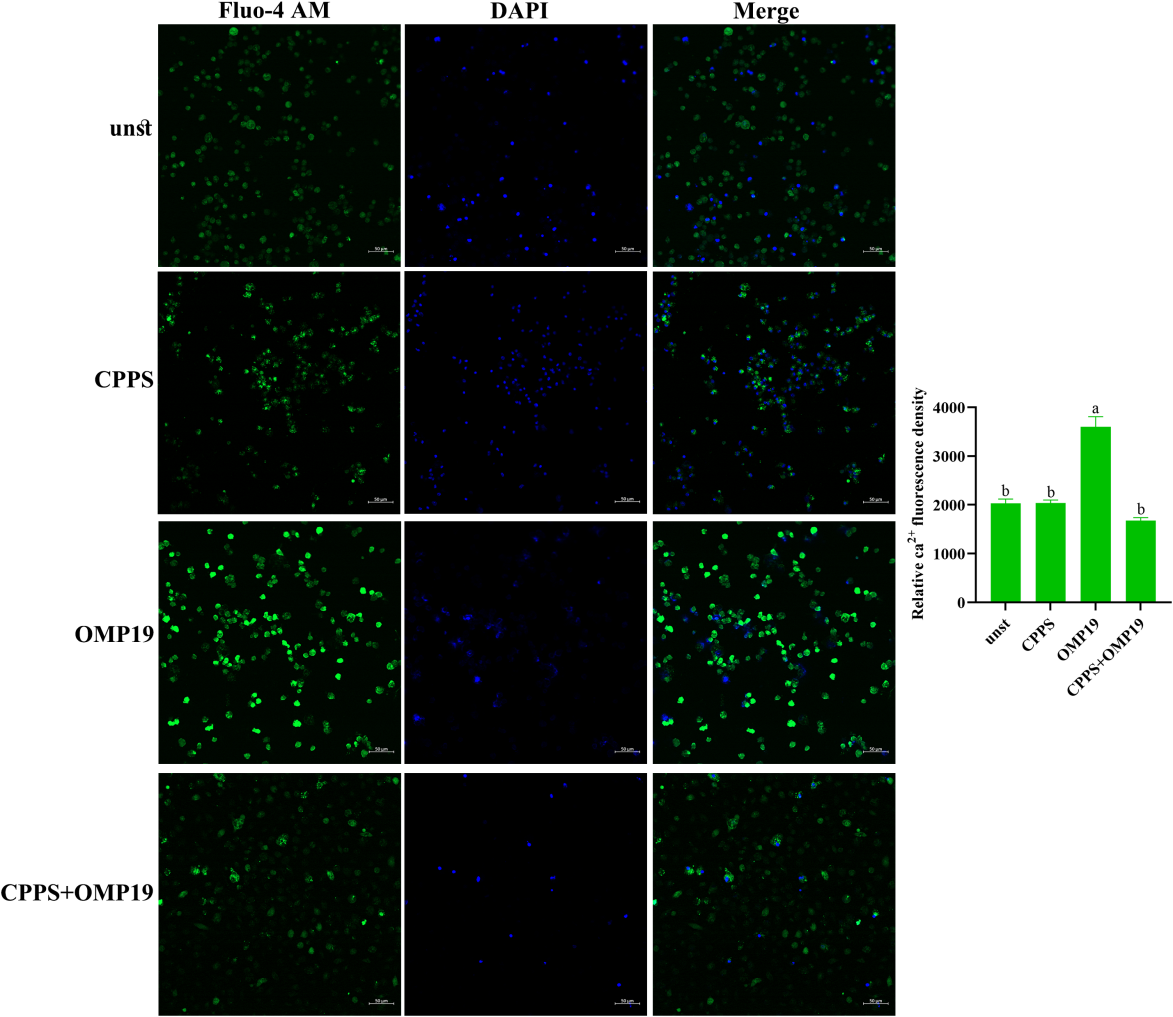
CPPS inhibited the inward flow of Ca^2+^ in macrophages induced by OMP19. Mouse peritoneal macrophages were pretreated with 50 µg·mL^-1^ CPPS for 12 h, followed by 5 µg·mL^-1^ OMP19 for 12 h. Ca^2+^ influx was significantly reduced in the CPPS+OMP19 co-treatment group compared with the OMP19 group (*P* < 0.05). Fluorescence intensity was measured using confocal microscopy. n = 3.

Ca^2+^, as a key second messenger, mediates the formation and maintenance of intercellular adhesion junctions by regulating calmodulin dimerization (37). The effect of CPPS on OMP19-induced intracellular Ca^2+^ flux in macrophages was analyzed using the Fluo-4 AM fluorescent probe (**Figure 6**, *P* < 0.001). Macrophages were pretreated with CPPS (50 µg·mL^-1^) for 12 h, followed by stimulation with OMP19 (5 µg·mL^-1^) for 12 h. As shown in **Figure 6**, OMP19 stimulation induced a significant increase in intracellular calcium flux, as quantified by Fluo-4 AM fluorescence and evidenced by elevated fluorescence intensity (*P* < 0.001). CPPS pretreatment effectively restored calcium levels to baseline, demonstrating its regulatory capacity in macrophage calcium homeostasis.

### 3.7 BAPTA-AM reversed the inhibitory impact of CPPS on cell adhesion function

**Figure 7:**
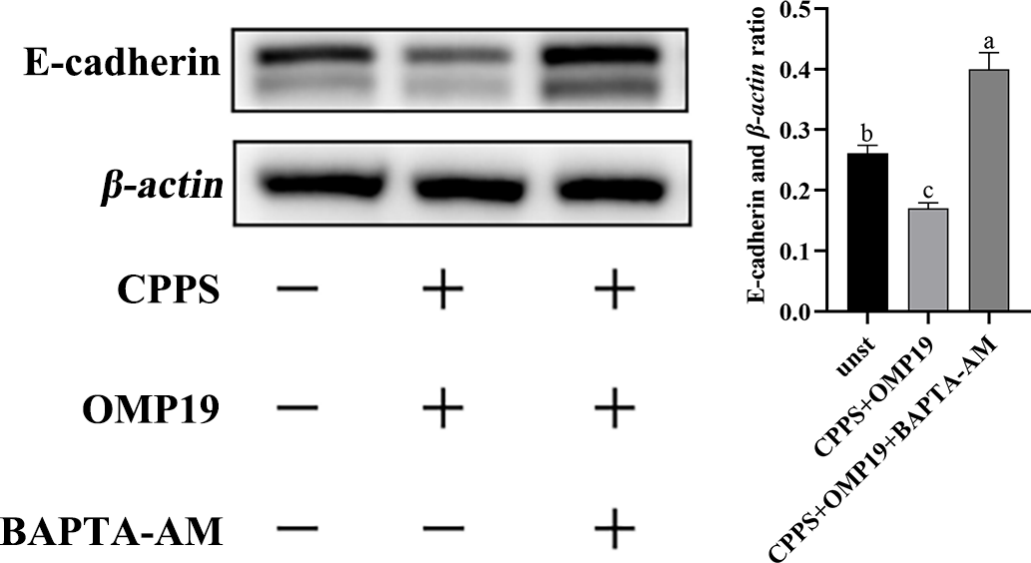
BAPTA-AM reversed the inhibitory impact of CPPS on cell adhesion function. Mouse peritoneal macrophages were pretreated with 100 µM BAPTA-AM for 1 h, followed by CPPS (50 µg·mL^-1^ for 12 h) and OMP19 (5 µg·mL^-1^ for 8 h). E-cadherin expression was analyzed by Western blot. *β-actin* was used as a loading control. Grayscale values were measured using ImageJ software. n = 3.

BAPTA-AM, a well-known membrane-permeable Ca^2+^ chelator, prevents cell injury by alleviating intracellular calcium overload (38). To investigate whether CPPS regulates the expression of E-cadherin by modulating Ca^2+^ influx and thereby affects macrophage adhesion, BAPTA-AM was used for verification. The results showed that the expression level of E-cadherin in the BAPTA-AM pretreatment group was significantly higher than that in the CPPS and OMP19 combined treatment group (*P* < 0.01, **Figure 7**), indicating that BAPTA-AM pretreatment reversed CPPS-induced downregulation of E-cadherin protein expression. These findings further demonstrate that CPPS most likely inhibits E-cadherin-mediated cell adhesion by reducing calcium ion levels in macrophages.

### 3.8 CPPS inhibited the activation of signaling pathways in macrophages induced by OMP19

**Figure 8:**
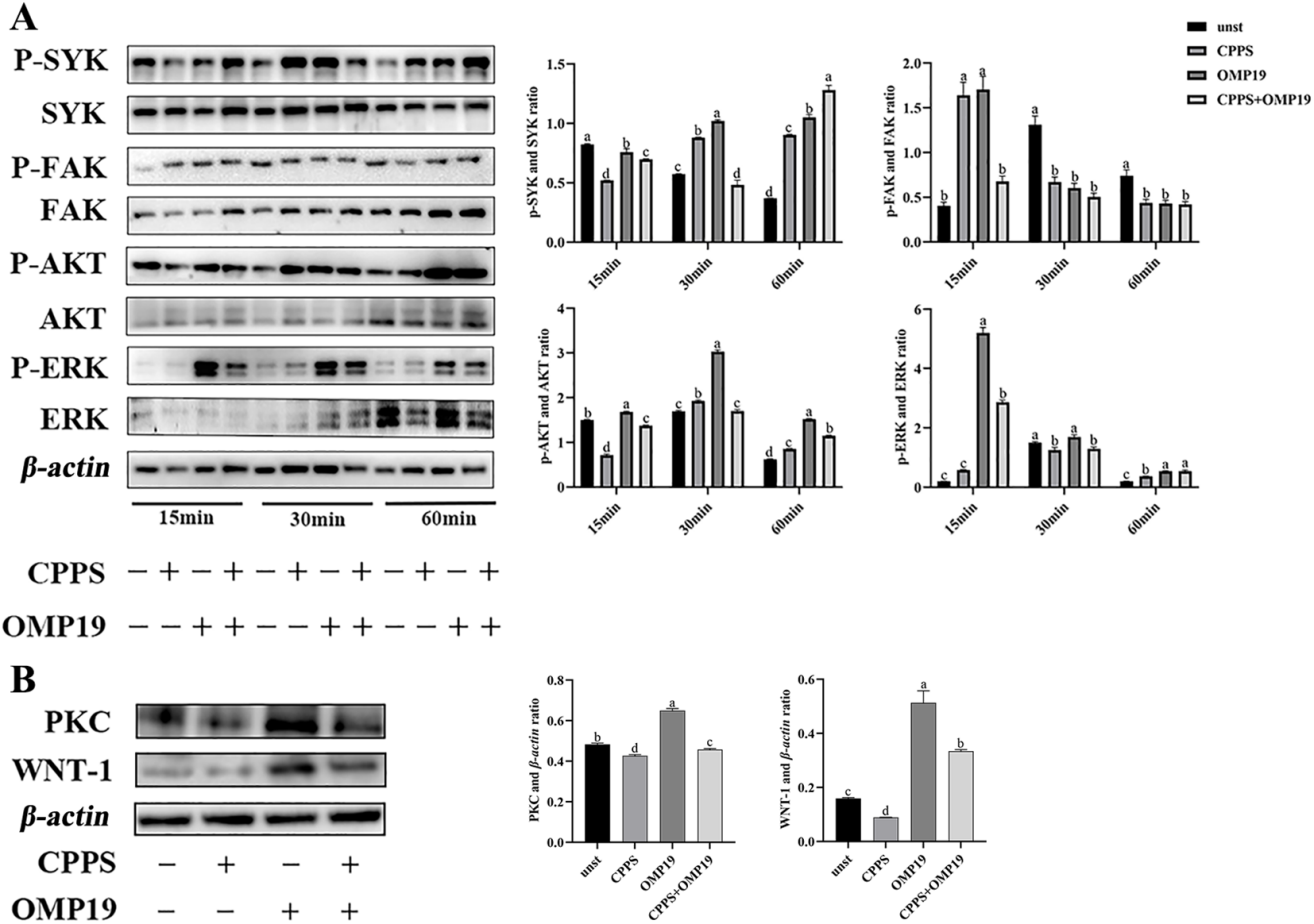
CPPS inhibited the activation of signaling pathways in macrophages induced by OMP19. **(A)** Mouse peritoneal macrophages were pretreated with 50 µg·mL^-1^ CPPS for 12 h, followed by 5 µg·mL^-1^ OMP19 for 15 min, 30 min, and 60 min. Phosphorylation and non-phosphorylation levels of ERK, AKT, FAK, and SYK were evaluated by Western blot. **(B)** Mouse peritoneal macrophages were pretreated with 50 µg·mL^-1^ CPPS for 12 h, followed by 5 µg·mL^-1^ OMP19 for 8 h. Expression levels of PKC and WNT-1 were measured by Western blot. *β-actin* was used as a loading control. Grayscale values were measured using ImageJ software. n = 3.

To investigate the effect of CPPS on OMP19-induced macrophage adhesion function, we examined the activation levels of the SYK/AKT/FAK, ERK, PKC, and WNT-1 signaling pathways. The results showed that CPPS significantly downregulated the phosphorylation levels of SYK/AKT/FAK at the corresponding time points in OMP19-stimulated cells (as shown in **Figure 8A**), suggesting that CPPS may hinder the cell adhesion process. At 15 and 30 min after OMP19 stimulation, ERK phosphorylation was significantly upregulated compared with the control group. In contrast, the CPPS-pretreated OMP19-stimulated group exhibited a marked downregulation of ERK phosphorylation compared with the OMP19-stimulated group (**Figure 8A**). These results suggest that CPPS may reduce the expression of macrophage inflammatory factors by inhibiting activation of the ERK signaling pathway. **Figure 8B** shows that the CPPS-pretreated OMP19-stimulated group exhibited significant downregulation of PKC and WNT-1 activation compared with the group stimulated with OMP19 alone. Classical PKC activation requires Ca^2+^ co-signaling, and these results are consistent with those obtained for Ca^2+^ influx (**Figure 6**).

### 3.9 Effects of *ATP2A1* knockdown on the CPPS-mediated attenuation of OMP19-induced inflammatory activation and upregulation of E-cadherin and paxillin expression in THP-1 cells

**Figure 9:**
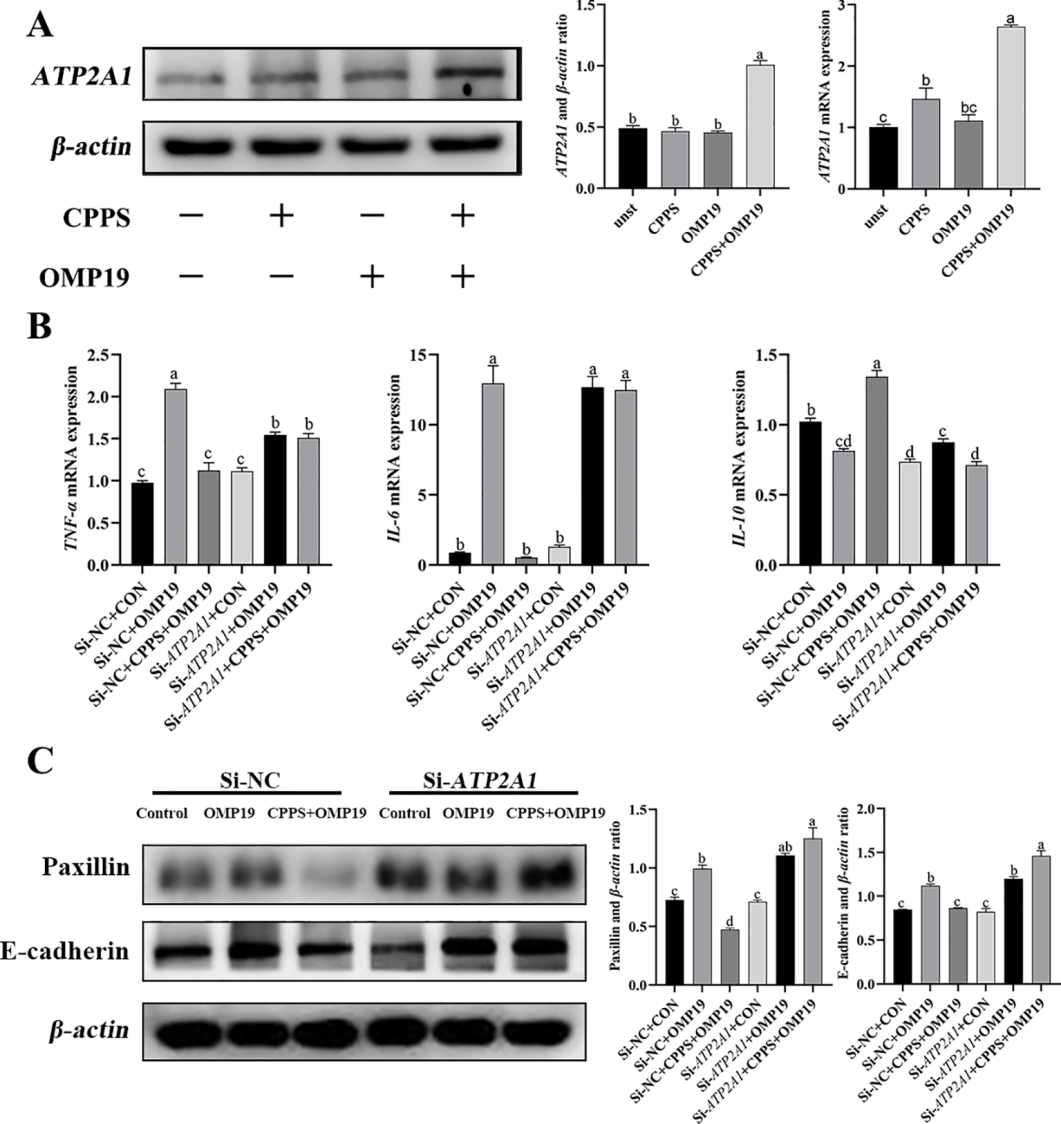
Effects of *ATP2A1* knockdown on CPPS alleviating OMP19-induced inflammatory activation, while increasing E-cadherin and paxillin of THP-1 cells. **(A)** THP-1 cells were pretreated with 50 µg·mL^-1^ CPPS for 12 h, followed by stimulation with 5 µg·mL^-1^ OMP19 for another 8 h. Western blot was performed to detect the expression of *ATP2A1*, with *β-actin* used as the loading control. THP-1 cells were pretreated with 50 µg·mL^-1^ CPPS for 12 h and subsequently exposed to 5 µg·mL^-1^ OMP19 for 12 h. RT-qPCR was conducted to measure the mRNA expression level of *ATP2A1* in macrophages. **(B)** After *ATP2A1* knockdown, THP-1 cells were pretreated with 50 µg·mL^-1^ CPPS for 12 h, followed by treatment with 5 µg·mL^-1^ OMP19 for another 12 h. The mRNA expression levels of *TNF-α*, *IL-6*, and *IL-10* were detected by RT-qPCR. **(C)** After *ATP2A1* knockdown, THP-1 cells were pretreated with 50 µg·mL^-1^ CPPS for 12 h, followed by stimulation with 5 µg·mL^-1^ OMP19 for another 8 h. Western blot was performed to detect the protein expression levels of E-cadherin and paxillin, with *β-actin* used as the loading control. The grayscale values of protein bands were analyzed using ImageJ software. Grouping: Si-NC+CON, transfected with negative control siRNA; Si-NC+OMP19, transfected with negative control siRNA + OMP19 stimulation; Si-NC+CPPS+OMP19, transfected with negative control siRNA + CPPS + OMP19 stimulation; Si-*ATP2A1*+CON, transfected with *ATP2A1* siRNA control group; Si-*ATP2A1*+OMP19, transfected with *ATP2A1* siRNA + OMP19 stimulation; Si-*ATP2A1*+CPPS+OMP19, transfected with *ATP2A1* siRNA + CPPS + OMP19 stimulation. n = 3. Lowercase letters a, b, c and d in the figures indicate statistical significance. Different letters mean significant differences between groups, while the same letter indicates no significant difference.

*ATP2A1* encodes sarcoplasmic/endoplasmic reticulum calcium ATPase 1 (SERCA1), a key regulator responsible for the reuptake of cytoplasmic Ca^2+^ into the endoplasmic reticulum, which plays a vital role in maintaining cellular calcium homeostasis (39, 40). Western blotting and RT-qPCR were performed to detect the expression level of *ATP2A1* (**Figure 9A**). CPPS treatment significantly upregulated *ATP2A1* expression in macrophages, which was consistent with the transcriptome sequencing results. To further elucidate the molecular mechanism by which *ATP2A1* regulates immune responses in macrophages, siRNA-mediated knockdown assays were conducted for subsequent functional verification. RT-qPCR results (**Figure 9B**) revealed that OMP19 stimulation markedly upregulated the mRNA levels of *TNF-α* and *IL-6* while downregulating *IL-10* expression. CPPS treatment significantly reduced the elevated *TNF-α* and *IL-6* levels and restored the suppressed *IL-10* expression (*P* < 0.01). Following *ATP2A1* knockdown, CPPS failed to further inhibit OMP19-induced upregulation of *TNF-α* and *IL-6* and could not reverse the decrease in *IL-10* expression, resulting in a complete loss of its anti-inflammatory effects. Meanwhile, Western blotting showed (**Figure 9C**) that following *ATP2A1* knockdown, CPPS could not decrease OMP19-induced downregulation of E-cadherin and paxillin expression. These results confirm that the immunomodulatory effects of CPPS depend on *ATP2A1*-mediated regulation of cellular calcium homeostasis.

## 4 Discussion

HMGB1 is a late-stage pro-inflammatory mediator that is released in various models of cell death and injury, and it plays a critical role in mediating inflammation triggered by cell death-associated responses (41). *Brucella* infection of trophoblasts has been shown to trigger HMGB1 release into the extracellular space (42), contributing to tissue injury in reproductive organs (43). In this study, CPPS significantly reduced OMP19-induced HMGB1 expression in uterine tissue, suggesting its ability to mitigate HMGB1-driven inflammatory damage. Similar anti-inflammatory effects of CPPS have been reported in LPS- and *E. coli*-induced lung injury models (28), supporting a broader protective role against inflammatory tissue injury.

Since CPPS can alleviate OMP19-mediated uterine injury in mice, mouse peritoneal macrophages were selected as the research object for transcriptome sequencing to further elucidate the regulatory mechanism of CPPS. Transcriptomic profiling revealed that CPPS modulated gene expression patterns enriched in focal adhesion and calcium signaling pathways, suggesting functional reprogramming of macrophages under inflammatory challenge. Although E-cadherin is a classic epithelial marker, accumulating evidence has demonstrated that it regulates macrophage fusion, multinucleated giant cell formation, and participates in macrophage adhesion and migration (44, 45). Meanwhile, paxillin also mediates cellular adhesion, migration, and cytoskeletal remodeling (45, 46). Therefore, the expression alterations of these molecules in this study mainly reflect changes in macrophage adhesion rather than epithelial tissue remodeling. Furthermore, the present study confirms that CPPS can significantly inhibit OMP19-induced upregulation of adhesion molecules such as E-cadherin and paxillin and alleviate excessive intracellular Ca^2+^ influx. Elrashedy et al. showed that Omp25 and Omp31 can promote bacterial adhesion and intracellular invasion (47). Thus, the downregulation of adhesion molecules by CPPS may effectively limit the colonization of *Brucella* in macrophages.

Admittedly, OMP19 stimulation cannot represent the entire inflammatory process of *Brucella*-infected macrophages, which is a limitation of this study. In the future, live *Brucella* will be used to further investigate the regulatory effects of CPPS, thereby enhancing its translational value in the clinical treatment of *Brucella* infection. *ATP2A1*, as a core member of the endoplasmic reticulum Ca^2+^-ATPase family, is essential for maintaining cellular Ca^2+^ homeostasis (48–50). Intracellular Ca^2+^ flux plays a dual role in host defense. Moderate Ca^2+^ increases promote phagosome-lysosome fusion and pathogen clearance (51), whereas sustained calcium overload can impair mitochondrial function, reduce ATP and ROS production, and exacerbate inflammation via NF-κB and NLRP3 activation (52, 53). OMP19 markedly increased macrophage Ca^2+^ influx in this study, potentially contributing to excessive inflammatory activation. CPPS normalized calcium levels, suggesting its ability to prevent calcium-mediated macrophage dysfunction. BAPTA-AM reversed the inhibitory effect of CPPS on cell adhesion function, supporting a mechanism involving modulation of Ca^2+^-dependent adhesion processes. After knockdown of *ATP2A1*, the anti-inflammatory effect of CPPS was completely abolished, and it failed to reverse the OMP19-induced downregulation of adhesion molecule expression.

At the signaling level, CPPS inhibited OMP19-induced phosphorylation of SYK, FAK, AKT, and ERK, all of which contribute to adhesion complex assembly and inflammatory mediator production (16, 54, 55). Additionally, CPPS suppressed PKC activation and WNT-1 expression, pathways known to integrate calcium signaling and cytoskeletal reorganization (20, 21). By targeting multiple nodes in these signaling cascades, CPPS may simultaneously restrain excessive adhesion and downregulate pro-inflammatory outputs. CPPS suppressed pro-inflammatory factors (*TNF-α*, *IL-6*, *CXCL-10*, and *MIP-1α*) while enhancing anti-inflammatory *IL-10* production. Similar to *Ganoderma lucidum* polysaccharides, which reduce the secretion levels of *TNF-α* and *IL-6* in mouse liver (56), CPPS also exerts anti-inflammatory effects against OMP19-mediated inflammation. This may suggest that polysaccharides derived from traditional Chinese medicine generally possess anti-inflammatory properties.

This study has several limitations. Pharmacokinetic analysis of CPPS was not performed, and its *in vivo* metabolic profile and accumulation characteristics remain unclear. Intraperitoneal injection was adopted in animal experiments, which differs greatly from oral administration in clinical practice. Thus, the experimental dosage cannot be directly extrapolated, and the safe clinical dosage and administration frequency in humans require further investigation.

## 5 Conclusion

CPPS mitigates OMP19-induced uterine injury by modulating macrophage adhesion and calcium-dependent signaling. Specifically, CPPS suppresses excessive Ca^2+^ influx, downregulates E-cadherin and paxillin expression, and inhibits SYK/FAK/AKT and ERK phosphorylation, while attenuating PKC and WNT-1 activation. These effects collectively reduce pro-inflammatory cytokine production and enhance anti-inflammatory *IL-10* expression, thereby limiting tissue damage. Further *ATP2A1* knockdown experiments demonstrated that the effects of CPPS in alleviating OMP19-induced inflammatory responses are dependent on the regulation of *ATP2A1* expression.

## Data Availability

The data presented in the study are deposited in the GEO repository, accession number GSE314097.
